# Testing for Basins of Wada

**DOI:** 10.1038/srep16579

**Published:** 2015-11-10

**Authors:** Alvar Daza, Alexandre Wagemakers, Miguel A. F. Sanjuán, James A. Yorke

**Affiliations:** 1 Nonlinear Dynamics, Chaos and Complex Systems Group, Departamento de F´ısica, Universidad Rey Juan Carlos, Móstoles, Madrid, Tulipán s/n, 28933, Spain; 2 University of Maryland, College Park, Maryland 20742, USA

## Abstract

Nonlinear systems often give rise to fractal boundaries in phase space, hindering predictability. When a single boundary separates three or more different basins of attraction, we say that the set of basins has theWada property and initial conditions near that boundary are even more unpredictable. Many physical systems of interest with this topological property appear in the literature. However, so far the only approach to study Wada basins has been restricted to two-dimensional phase spaces. Here we report a simple algorithm whose purpose is to look for the Wada property in a given dynamical system. Another benefit of this procedure is the possibility to classify and study intermediate situations known as *partially* Wada boundaries.

Sometimes a physical property can be labeled with different discrete values depending on parameters. Suppose a space has 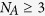 disjoint regions *S*_*j*_ where each *S*_*j*_ represents a different value or state. For example the sets might be basins of attraction in the phase space of a dynamical system. Numerical and experimental investigations of the property in question can be intricate when the boundaries between the sets are fractal. It may be difficult to predict the state transition of the system as the initial condition is disturbed. This unpredictability increases when the boundaries of *S*_*j*_ are not only fractal but also possess the *Wada property*; that is, each point on the boundary of any of these regions is in fact on the boundary of all of them. In other words, the sets share the same boundary.

This situation emerges for a variety of systems of high interest in physics such as the basins of the forced damped pendulum^1^, escapes in tokamaks^2^, dyes in open hydrodynamical flows^3^, the Duffing equation with a periodic forcing^4^, the Hénon-Heiles system^5^, and the use of Newton’s method to find complex roots^6^.

The problem first arose as an investigation of regions in the plane: Can three or more open, disjoint, connected regions *S*_*j*_ in the plane have the same boundary? This question was answered in the affirmative by L.E.J. Brouwer in^7^. In^8^ K. Yoneyama gave an example that he attributes to *Mr. Wada*, his Ph.D. supervisor, Takeo Wada. Hocking and Young^9^ used the term *lakes of Wada*, a pun on water, so it was natural to extend the wordplay to dynamical systems by introducing the term *basins of Wada*^10^. The papers^11^,^12^ applied this concept to dynamical systems and devised a method to identify Wada basins. It is remarkable how improbable the original topological examples appeared, seeming unrelated to anything real, and yet surprisingly appear to be common in dynamical systems including analytic systems like the forced damped pendulum.

The Nusse-Yorke (NY) method^12^ observes that when the unstable manifold of a boundary saddle point *q* crosses three or more different basins, then the point *q* is a Wada point, as is every point in the stable manifold of *q* and in its closure. It can be difficult to show that the closure of that stable manifold is the entire boundary. The procedure for carrying that out is based on a concept called *basin cells*, which requires detailed knowledge of stable and unstable manifolds. The NY method is an adequate approach for basins in two dimensional phase spaces. However, the method is not suitable in many cases.

The Wada property appears in diverse situations such as the parameter space of the Hénon map^13^ or the one-dimensional phase space of a competition model in ecology^14^. We can also find experimental and theoretical examples where the Wada property seems very likely to be present but the absence of a proper method of characterization prevents its study^15^,^16^. We present a special case where the basins of the dynamical system share their boundaries, but the different basins are not connected. These *disconnected* Wada basins can be illustrated as follows. Figure 1(a): divide a disk in six sectors and color three alternate sectors with different colors; Fig. 1(b): divide each empty sector into three sectors and color the central one with the color that is not at the left nor at the right; Fig. 1(c): repeat the second step indefinitely until filling the whole disk. The boundary of the different colors displays the Wada property by construction, but the basins are not connected; indeed the set of Wada points on the boundaries possesses a Cantor set structure. There is a rich literature documenting systems with disconnected Wada sets: the experiment of light scattered by reflecting balls^17^, the Newton method to find complex roots^6^, chaotic scattering in more than two dimensions^18^, etc.

## A Grid Approach

We propose a simple method, straightforward to implement, to test the Wada property in all kind of systems. Furthermore, our method/terminology allows us to classify intermediate situations where only some basins and some of the boundary present the Wada property, which receives the name of *partially* Wada basins^19^,^20^.

We first need some assumptions. We will discuss the notation for the case of a two dimensional space though it is easily adapted to other cases.

1. There is a bounded region Ω containing 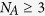 disjoint regions *S*_*j*_ where *j* = 1, …, *N*_*A*_.
2. There is a rectangular grid *G* covering Ω. We typically use a 1000 × 1000 grid. Hence Ω is covered by a set 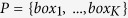 of grid boxes (whose interiors do not intersect each other). Here *K* would be 10^6^ for that usual grid.
3. For each point *x* in Ω, it is possible to determine to which set *S*_*j*_ it belongs to. In other words, there is a function *C* with *C*(*x*) = *j* if 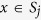 and *C*(*x*) = 0 if *x* is in none of the sets *S*_*j*_. If the sets are basins, the trajectory for each 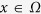 leads to an atractor labelled by *C*(*x*). Notice that we do not impose different labels for each attractor. It is possible to merge several attractors into the same category. For any rectangular box denoted as *box* we define *C*(*box*) = *C*(*x*) where *x* is the point at the center of *box*. If it does not go to an attractor in our collection of numbered attractors, then *C*(*box*) = 0, such events are reported at the end of the run. For convenience we will refer to this numerical value *C* as the *color* of the grid box. Of course other points in the same box might lead to different attractors.
4. We define *b*(*box*_*j*_) to be the collection of grid boxes consisting of *box*_*j*_ and all the grid boxes that have at least one point in common with *box*_*j*_, so in dimension two, *b*(*box*_*j*_) is a 3 × 3 collection of boxes with *box*_*j*_ being the central box.
5. For each *box*_*j*_, we determine the number of different (non-zero) colors in *b*(*box*_*j*_) and write *M*(*box*_*j*_) for that number.
6. In each *box*_*j*_ with 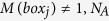, that is, which is not in the interior nor in the Wada boundary, we accomplish the following procedure. We select the two closest boxes in *b*(*box*_*j*_) with different colors and trace a line segment between them. We compute the color of the middle point of the segment. In case that the color newly computed completes all colors inside *b*(*box*_*j*_), then *M*(*box*_*j*_) = *N*_*A*_ and the algorithm stops. Otherwise, we compute two new points: one in the middle of the leftmost and central point, and another in the middle of the rightmost and central point. In the second step, four points interspersed with the previous five points would be calculated. In the third step, we would compute eight points interspersed with the previous nine. The procedure keeps on until *M*(*box*_*j*_) = *N*_*A*_ or the number of calculated points in that segment reaches some maximum value previously set up. A major computational advantage of this method is that the refinement is made in a one dimensional subspace (the segment linking the two points), no matter the dimension of Ω.
7. Next we define *G*_*m*_ to be the set of all the original grid boxes *box*_*j*_ for which *M*(*box*_*j*_) = *m*.

For *m* = 1, all the boxes inside the ball *b*(*box*_*j*_) have the same color as they all lead to the same attractor. In fact *G*_*1*_ represents points that are in the interior of a basin. A grid box *box*_*j*_ is in the set *G*_*2*_ (boundary of two) if there are two different colors inside the ball *b*(*box*_*j*_), a grid box is in the set *G*_3_ (boundary of three) if there are three different colors inside the ball and so forth. To account for the evolution of these sets as the algorithm progresses, we call 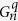 the set *G*_*n*_ at step *q*.

Then, we will say that the system is Wada if 
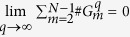
. This simply means that the grid boxes are either in the interior *G*_1_ or in the Wada boundary 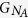 after a sufficient number of steps *q*.

The basic idea underlying the whole process is that if three basins are Wada, then it is always possible to find a third color between the other two colors (similar reasoning can be done for Wada basins with more than three colors). Notice also that if a boundary separates two basins we will only see those two basins at all resolutions.

**Figure 2:**
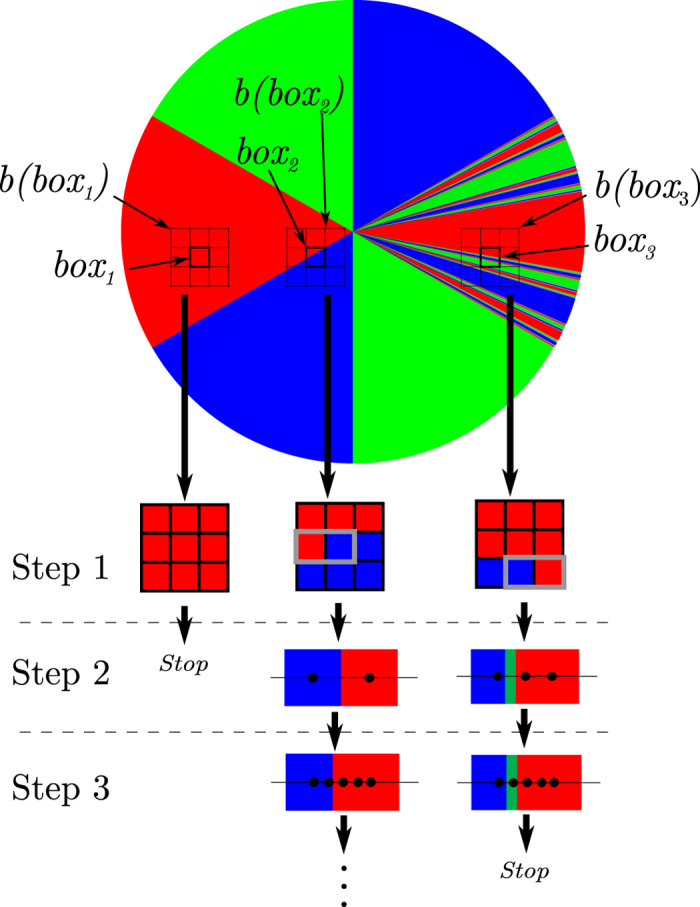
Sketch of the method. We set up a grid of boxes *box*_*j*_ covering the whole disk. The center point of each box defines its color. In the first step, we see that *box*_1_ belongs to the interior because its surrounding 8 boxes have the same color. On the other hand, *box*_2_ and *box*_3_ are in the boundary of two attractors, i.e., they are adjacent to boxes whose color is different. In the next step the algorithm classifies *box*_2_ still in *G*_2_ (boundary of two), while *box*_3_ is now classified in *G*_3_ (boundary of three). Ideally the process would keep on forever redefining the sets *G*_1_, *G*_2_ and *G*_3_ at each step, though in practice we can impose some stopping condition. This plot constitutes an example of *partially* Wada basins.

To illustrate the iterative process we represent in Fig. 2 our example of a partial Wada set and compute the boundary set for three grid boxes *box*_1_, *box*_2_, and *box*_3_ on a regular rectangular grid forming a partition *P*^0^ of the phase space. The first iteration for *box*_1_ shows that it belongs to the interior region 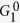, as the eight boxes surrounding it have the same color. At this point, we can consider *box*_1_ in 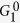 without refining the partition. The second, *box*_2_, lies in the boundary of two sets because two different colors are found in its ball. The successive iterations of the algorithm classify *box*_2_ into *G*_2_. A different situation arises for *box*_3_. The first iteration classifies *box*_3_ 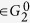 because only two colors are found in its ball. But as we increase the resolution, *box*_3_ turns out to be in a boundary of three basins 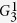.

In order to decide whether a system is Wada, not Wada, or presents an intermediate situation, we can count the number of boxes belonging to the boundary of *m* different basins. For that purpose we can define a useful parameter


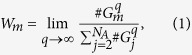


where 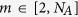. This parameter 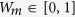 takes a value zero if the system has no grid boxes that are in the boundary separating *m* basins and it takes a value one if all the boxes in the boundary separate *m* basins. Thus, if 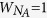 the system is said to be Wada. Partial Wada occurs when 0 < *W*_*m*_ < 1 with 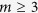. As we will see, *W*_*m*_ is also useful to test the global numerical convergence.

## Results

For all the results presented in this work, we use as initial partition a uniform grid of one million boxes, and the verification of the Wada property is subjected to that resolution. In order to illustrate the features of the described method, we will present an analysis of the results for the damped forced pendulum and the Newton method to find complex roots. They could be considered as paradigmatic examples of connected and disconnected Wada sets respectively. Nonetheless, we have also tested our method for the Duffing oscillator^4^, the Hénon-Heiles system^5^ and the magnetic pendulum^21^,^22^. In all of them, we have obtained values of *W*_3_ = 1 which means they all share the Wada property.

**Figure 3:**
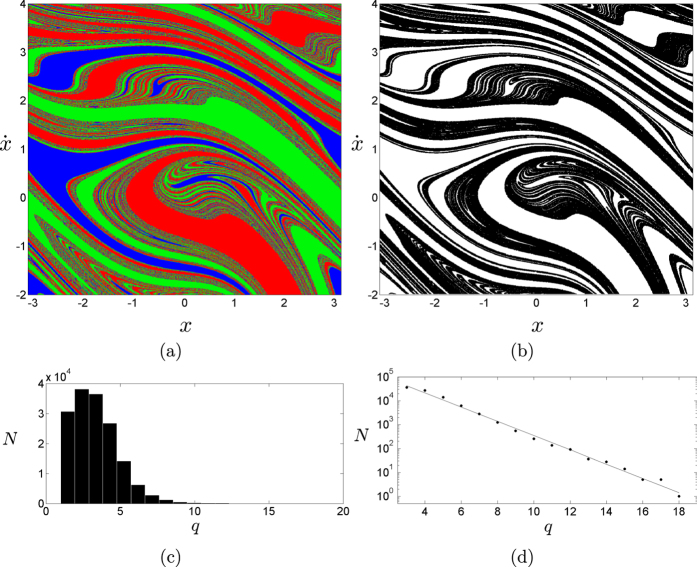
Forced damped pendulum. (**a**) Basins of attraction for the damped forced pendulum 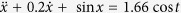. (**b**) All 1000 × 1000 boxes are labeled either in the interior (white) or in the boundary of the three basins (black). (**c**) Histogram showing the number of points *N* that take *q* steps to be classified as boundary of three. (**d**) After a maximum, there is an exponential decay of the computational effort related to the fractal structure of the basins. The log-plot reflects this tendency.

The first system we will analyze is the forced damped pendulum defined by 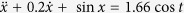, which constitutes a paradigmatic system with connected Wada basins^1^. After applying our method to the basin of attraction of Fig. 3(a), we find that all the boxes lie either in the boundary of the three basins or in the interior within the resolution of the method (see Fig. 3(b)). The histogram of Fig. 3(c) reflects the computational effort needed to test the Wada property in this system^23^. It shows that most of the points take less than five iterations to be labeled into the set boundary of three. Most importantly, the fractal structure of the boundary allows fast computation: as the number of steps *q* grows the number of remaining points *N* decreases exponentially (see Fig. 3(d)). At the end of the process, we find that *W*_3_ = 1 which implies that the system is Wada.

**Figure 4:**
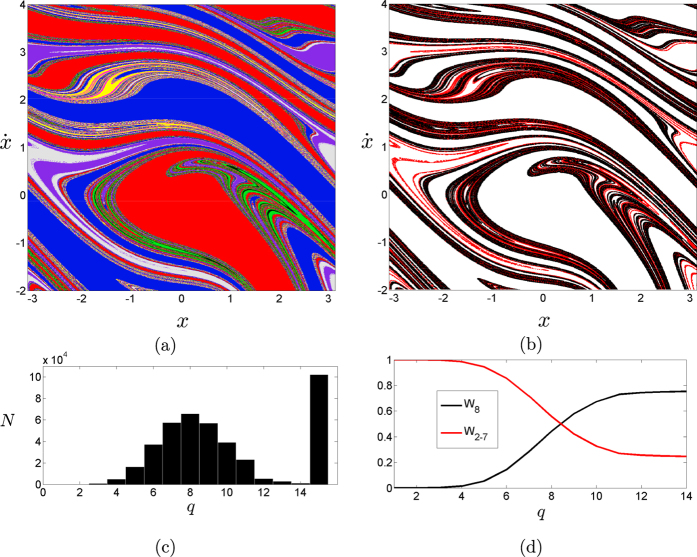
Forced damped pendulum with eight basins. (**a**) The damped forced pendulum with parameters 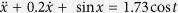 shows eight basins of attraction mixed intricately. (**b**) Some boxes are classified to be in the boundary of eight basins (black dots), but not all of them (red dots), which is a clear example of partial Wada. (**c**) The computational effort presents the usual shape for the Wada boundary, but the points which are not Wada keep refining indefinitely (bar at rightmost). Our algorithm works best in systems with the Wada property. (**d**) Evolution of the proportion of boxes in the Wada boundary (*W*_8_ in black) and proportion of boxes in a boundary which is not Wada (*W*_2−7_) as a function of the *q*-step. The convergence of *W*_8_ is used to determine the stopping rule.

A more challenging case arises when we increase the amplitude of the external forcing: 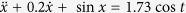. For these parameters the damped forced pendulum has at least eight attractors, and its basins are mixed complicatedly as shown in Fig. 4(a). When we apply our algorithm, we see that not all the boxes are classified as Wada (Fig. 4(b)). This is an example of partial Wada basins. The points in the boundary which are not Wada (points in *G*_2_ to *G*_7_ in our example) increases the computational effort of the algorithm (Fig. 4(c)). The reason is due to nature of the process, the algorithm stops computing when *M*(*box*_*j*_) = *N*_*A*_ for each box or reach the maximum of allowed steps.

In order to establish a stopping rule we can use the parameter *W*_*m*_: the algorithm stops when 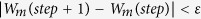, being *ε* a small positive number previously fixed (see Fig. 4(d)). Another option to deal with partial Wada is to merge some basins of attraction, considering two colors as only one single color for example. Making such a redefinition of the basins, we can say that the system is Wada if all the new basins share the same boundary (assuming we have at least three basins).

**Figure 5:**
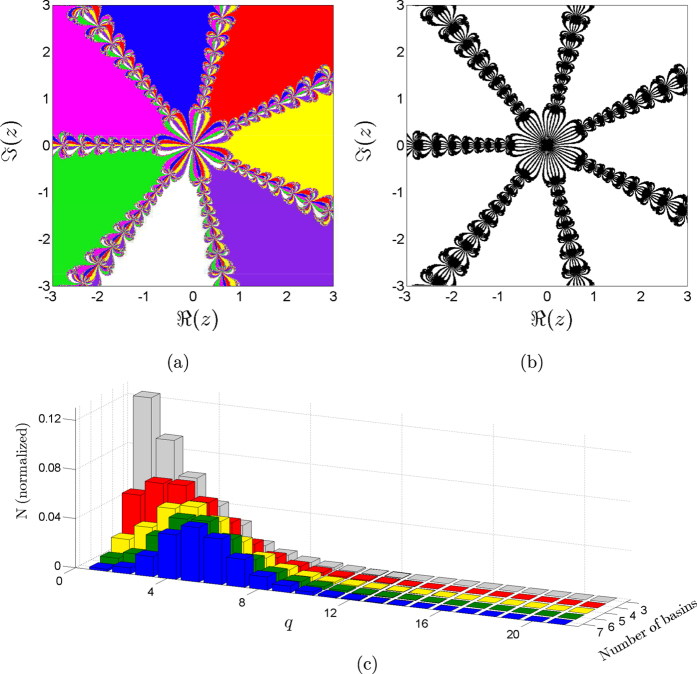
Newton method to find complex roots. (**a**) The map 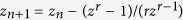 with *r* = 7 has seven basins of attraction with the disconnected Wada property. (**b**) All the boxes lie in the boundary of the seven basins or in the interior. (**c**) Computational effort as we vary *r* from 3 to 7. As the number of basins increases the maximum of the histograms shift to the right, that is, the more basins the larger the computational effort. The maximum number of steps *q* needed for any of these basins to be considered Wada is 21.

The second system under study is the Newton method to find complex roots, which provides examples of disconnected Wada sets. To find the roots of 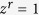, 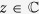 and 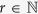, the Newton method iterates the map 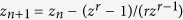. This map has *r* different fractalized and disconnected Wada basins corresponding to the complex roots of unity. In Fig. 5(a) we depict the basin for *r* = 7 and in Fig. 5(b) we see that the algorithm yields to *W*_7_ = 1, that is, all the boxes belong to the interior or to the boundary of the seven basins. Thus, our method can verify the Wada property for an arbitrary number of basins and for disconnected Wada sets too. An analysis of the computational effort for the Newton method varying *r* from 3 to 7 reflects that it grows with the number of basins (the maxima of the histograms in Fig. 5(c) shifts to the right as the number of basins increases).

## Discussion

Fractal Wada boundaries seem common in nonlinear systems. However they can be overlooked or misinterpreted with a simple fractal boundary. We can also have an intermediate situation such as partial Wada. Our algorithm shows that in a computationally affordable time, the Wada basins can be detected for a given grid precision. Furthermore it is possible to apply the technique to disconnected Wada sets, high-dimensional problems, experimental settings and partially Wada systems.

A simple key idea drives the search: on the line segment between two points of different basins there is always a point belonging to another basin if the boundary is Wada. The indicator *W*_*m*_ quantifies the Wada property and allows comparisons between basins. We can imagine for example a study of the measurement *W*_*m*_ as a function of a parameter of the system under study. We can also imagine an optimization algorithm to implement a heuristic search for Wada basins. We believe that this algorithm can constitute a powerful tool in the study of dynamical systems in general and of the Wada property in particular.

## Additional Information

**How to cite this article**: Daza, A. *et al.* Testing for Basins of Wada. *Sci. Rep.* **5**, 16579; doi: 10.1038/srep16579 (2015).

## Figures and Tables

**Figure 1:**
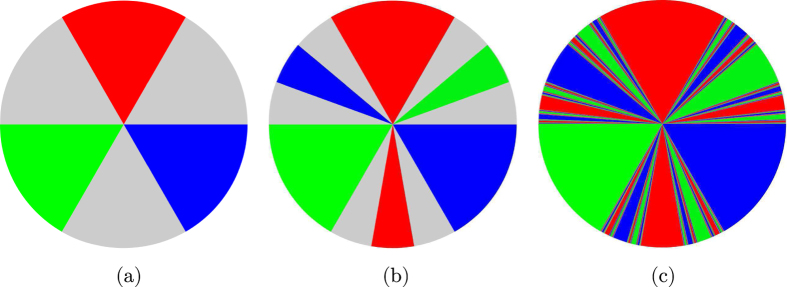
Disconnected Wada set. Three different stages to build a Wada set using three disconnected regions. The basins (colors) share the same boundary but each colored set is disconnected.

## References

[b1] Nusse H. E., Ott E. & Yorke J. A. Saddle-node bifurcations on fractal basin boundaries. Phys. Rev. Lett. 75(13), 2482–2485 (1995). 10059323 10.1103/PhysRevLett.75.2482

[b2] Portela J. S. E., Caldas I. L., Viana R. L. & Sanjuán M. A. F. Fractal and Wada exit basin boundaries in tokamaks. Int. J. Bifurc. Chaos 17(11), 4067–4079 (2007).

[b3] Toroczkai Z., Károlyi G., Péntek Á., Tél T., Grebogi C. & Yorke J. A. Wada dye boundaries in open hydrodynamical flows. Physica A 239(1), 235–243 (1997).

[b4] Aguirre J. & Sanjuán M. A. F. Unpredictable behavior in the Duffing oscillator: Wada basins. Phys. D 171(1), 41–51 (2002).

[b5] Aguirre J., Vallejo J. C. & Sanjuán M. A. F. Wada basins and chaotic invariant sets in the HÃ©non-Heiles system. Phys. Rev. E 64(6), 66208 (2001). 10.1103/PhysRevE.64.066208 11736269

[b6] Epureanu B. I. & Greenside H. S. Fractal basins of attraction associated with a damped Newton’s method. SIAM Rev. 40, 102–109 (1998).

[b7] Brouwer L. E. J. Zur analysis situs. Math. Ann. 68(3), 422–434 (1910).

[b8] Yoneyama K. Theory of continuous sets of points. Tokohu Math. J. 11(12), 43–158 (1917).

[b9] Hocking J. G. & Young G. S. Topology. Addison Wesley (Reading, Massachusetts) (1961).

[b10] Kennedy J. & Yorke J. A. Basins of Wada. Phys. D 51(1), 213–225 (1991).

[b11] Nusse H. E. & Yorke J. A. Fractal basin boundaries generated by basin cells and the geometry of mixing chaotic flows. Phys. Rev. Lett. 84(4), 626–629 (2000). 11017332 10.1103/PhysRevLett.84.626

[b12] Nusse H. E. & Yorke J. A. Wada basin boundaries and basin cells. Phys. D 90(3), 242 261.

[b13] Zhang Y. & Luo G. Unpredictability of the Wada property in the parameter plane. Phys. Lett. A 376(45), 3060–3066 (2012).

[b14] Vandermeer J. Wada basins and qualitative unpredictability in ecological models: a graphical interpretation. Ecol. Modell. 176(1), 65–74 (2004).

[b15] Gattobigio G. L., Couvert A., Georgeot B. & Guéry-Odelin D. Exploring classically chaotic potentials with a matter wave quantum probe. Phys. Rev. Lett. 107(25), 254104 (2011). 22243080 10.1103/PhysRevLett.107.254104

[b16] Gattobigio G. L., Couvert A., Reinaudi G., Georgeot B. & Guéry-Odelin D. Optically guided beam splitter for propagating matter waves. Phys. Rev. Lett. 109(3), 30403 (2012). 10.1103/PhysRevLett.109.030403 22861829

[b17] Sweet D., Ott E. & Yorke J. A. Topology in chaotic scattering. Nature 399(6734), 315–316 (1999).

[b18] Kovács Z. & Wiesenfeld L. Topological aspects of chaotic scattering in higher dimensions. Phys. Rev. E 63(5), 56207 (2001). 10.1103/PhysRevE.63.056207 11414990

[b19] Aguirre J., Viana R. L. & Sanjuán M. A. F. Fractal structures in nonlinear dynamics. Rev. Mod. Phys. 81(1), 333–386 (2009).

[b20] Zhang Y. & Luo G. Wada bifurcations and partially Wada basin boundaries in a two-dimensional cubic map. Phys. Lett. A 377(18), 1274–1281 (2013).

[b21] In the magnetic pendulum the fractality is lost at infinity^22^. Thus, this system does not have the Wada property from a mathematical point of view, but physically it does. Our algorithm classifies it as completely Wada system too.

[b22] Motter A. E., Gruiz M., Károlyi G. & Tél T. Doubly transient chaos: Generic form of chaos in autonomous dissipative systems. Phys. Rev. Lett. 111(19), 194101 (2013). 24266475 10.1103/PhysRevLett.111.194101

[b23] If only three colors are detected it is worth mentioning a straightforward simplification. Once the algorithm has chosen two points in a ball to look for the third color between them, one can use an oriented version of the method. Using this modification, the computational effort grows linearly with the number of steps *q* instead of exponentially.

